# Supplementary material for: A convolutional neural-network framework for modelling auditory sensory cells and synapses
Source: Commun Biol. 2021 Jul 1;4:827. doi: 10.1038/s42003-021-02341-5 (PMC8249591; doi:10.1038/s42003-021-02341-5)
Supplement: Supplementary file 1 — Supplementary Information [file 42003_2021_2341_MOESM1_ESM.pdf]

Supplementary information

A convolutional neural-network framework for modelling auditory sensory cells and synapses

Fotios Drakopoulos, Deepak Baby, Sarah Verhulst

Dept. of Information Technology, Ghent University, 9000 Ghent, Belgium

E-mail: * fotios.drakopoulos@ugent.be; deepakbabycet@gmail.com; s.verhulst@ugent.be

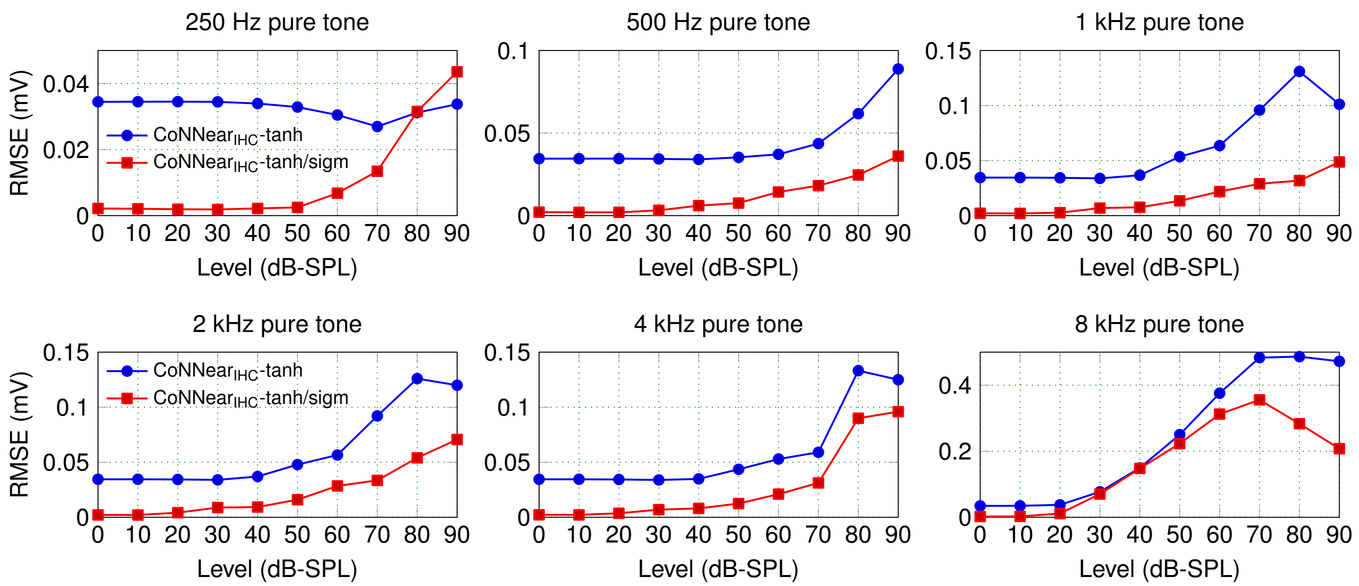

Supplementary Figure 1. Root-mean-square-errors (RMSEs) between simulated average IHC receptor potentials $\overline{V}_{\text{m}}$ of the reference and CoNNear IHC models (cf. Fig. 2). For each pure-tone stimulus, the tanh/sigmoid non-linearity combination resulted in significantly lower RMSEs across level compared to choosing a tanh nonlinearity for all layers. On average, the RMSE increased by a factor of nine for the 8-kHz excitation patterns. This decreased performance results from the frequency content of the speech training material (see Discussion).

Supplementary Table 1. CoNNear execution time for a full sentence. Comparison of the time required to process a full TIMIT sentence using each stage of the reference and CoNNear models on CPU and GPU. The sentence was zero-padded to account for the context effects of the CoNNear models, and the reported times correspond to the total time needed to transform the ∼4.1 s speech input to BM vibrations, IHC receptor potentials and ANF firing rates. The reference transmission-line cochlear model[60] and CoNNear$_{\text{cochlea}}$[66] simulate 401 and 201 N_{CF} frequency channels respectively, but fewer channels can be selected afterwards to demonstrate the effect of using lower frequency resolution (21-CF) or single-unit (1-CF) responses on the execution time of the IHC and ANF models. The single-channel CoNNear$_{\text{IHC-ANF}}$ models were used for all simulations to avoid large memory allocation, and population responses were simulated consecutively (channel by channel).

Model	Window	CPU (s)			GPU (ms)		
	(samples)	201-CF	21-CF	1-CF	201-CF	21-CF	1-CF
Cochlear model[60]	409,600	775.3194	-	-	-	-	-
CoNNear$_{\text{cochlea}}$[66]	81,920	2.9711	-	-	190.01	-	-
IHC model	409,600	36.0353	16.8936	15.6597	-	-	-
CoNNear$_{\text{IHC}}$	81,920	56.0590	6.0907	0.2776	1938.63	211.77	9.55
ANF$_{\text{H}}$ model	409,600	5.7521	3.5783	3.5204	-	-	-
CoNNear$_{\text{ANF}_\text{H}}$	81,920	17.7375	1.9434	0.0928	1473.17	157.84	6.98
ANF$_{\text{M}}$ model	409,600	5.8164	3.5594	3.5227	-	-	-
CoNNear$_{\text{ANF}_\text{M}}$	81,920	17.5934	1.9281	0.0988	1464.06	154.13	7.41
ANF$_{\text{L}}$ model	409,600	5.9176	3.6065	3.5267	-	-	-
CoNNear$_{\text{ANF}_\text{L}}$	81,920	16.0268	1.7366	0.1015	1074.07	121.61	5.48

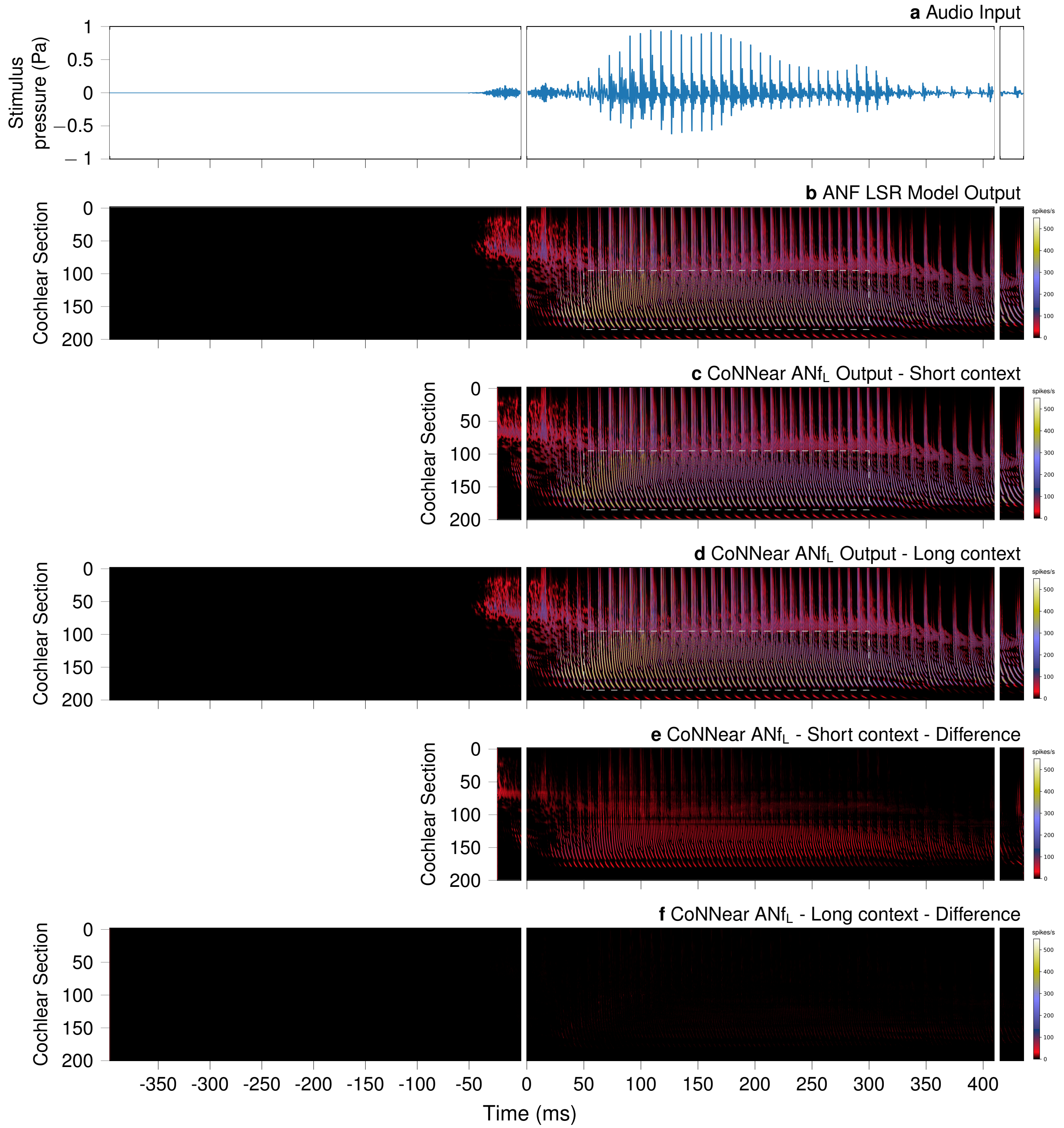

Supplementary Figure 2. Simulated ANF firing rates for a 8192-sample-long speech stimulus. The stimulus waveform is depicted in panel **a** and panels **b**-**d** depict the output firing rate (in spikes/s) of the reference ANF LSR model (**b**) and two CoNNear ANF LSR architectures, with a context of 256 samples (**c**) and 7936 samples (**d**) included on the left side of the input respectively. The audio stimulus was presented to the reference cochlear and IHC model and the simulated IHC receptor potential output was used to stimulate the three ANF models. The $N_{\text{CF}} = 201$ considered output channels are labeled per channel number: channel 0 corresponds to a CF of 112 Hz and channel 200 to a CF of 12 kHz. Furthermore, panel **e** shows the absolute difference between the outputs of the short-context architecture (**c**) and the reference ANF LSR model (**b**), while panel **f** shows the absolute difference between our final $\text{CoNNear}_{\text{ANF}_\text{L}}$ architecture (**d**) and the reference model (**b**).

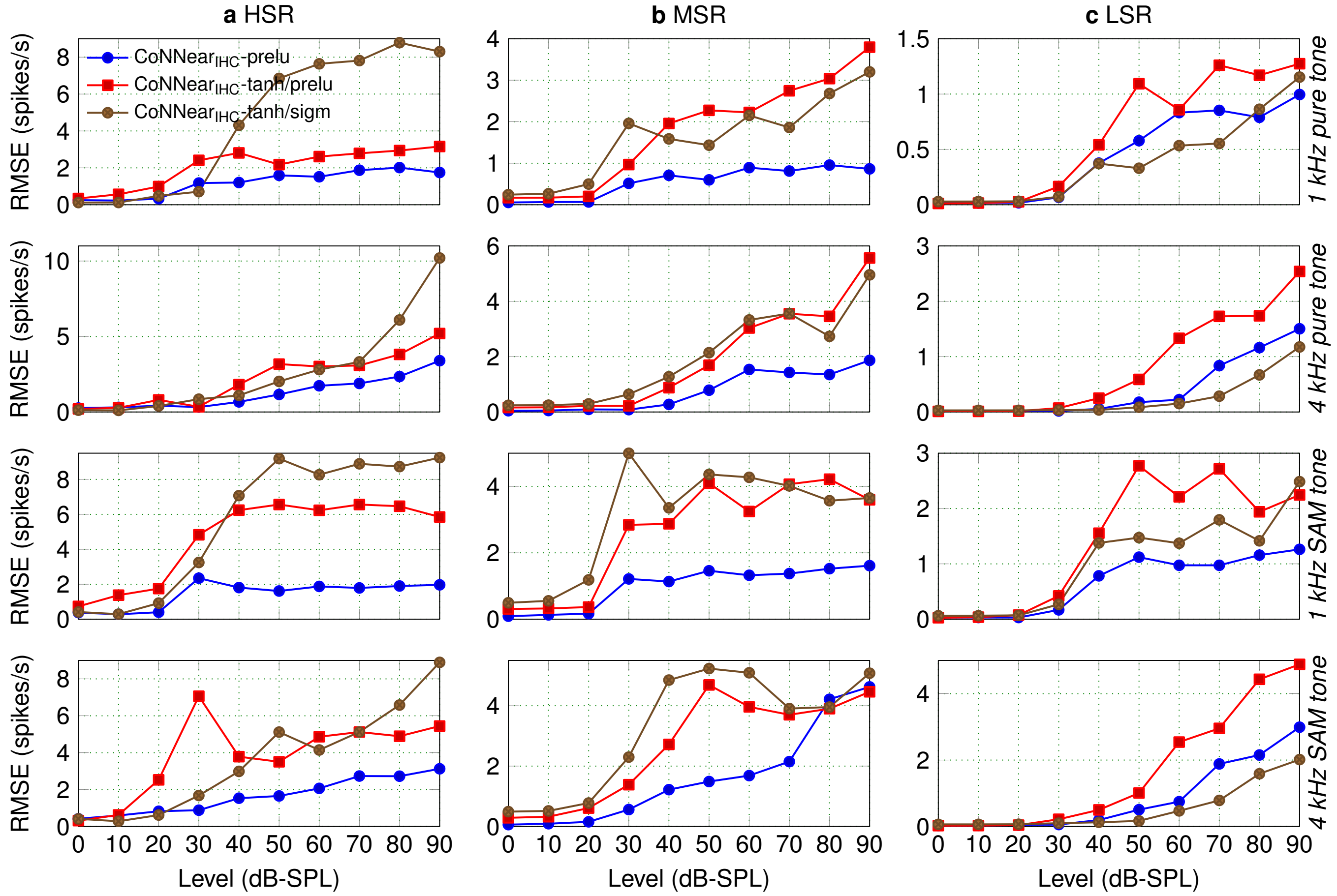

Supplementary Figure 3. RMSEs between simulated ANF firing rates of the reference and CoNNear ANF models, shown separately for each fiber type (cf. Fig. 5). For each of the tonal stimuli used for the ANF evaluation, the RMSE was computed across time for simulated firing rates of different levels. The selection of a PReLU non-linearity resulted in lower RMSEs for the HSR and MSR models (**a**,**b**), while the tanh/sigmoid architecture showed the best overall results for the LSR model (**c**).

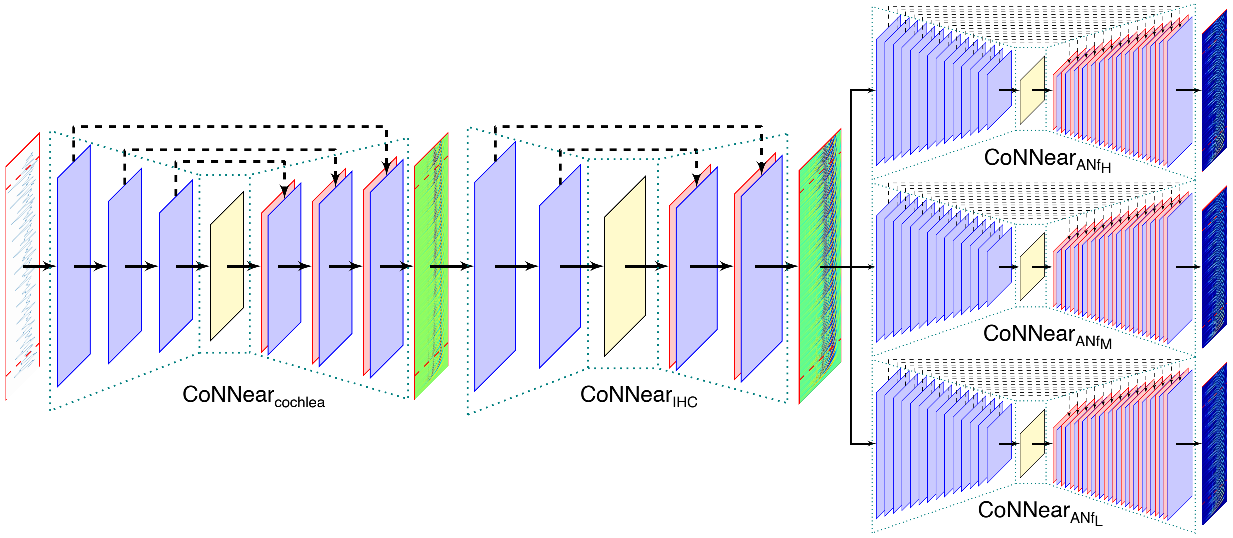

Supplementary Figure 4. CoNNear model of the auditory periphery. Acoustic stimuli can be transformed to IHC receptor potentials and ANF firing rates along the cochlear tonotopy and hearing range, after connecting the CoNNear cochlea[66], IHC and ANF modules together.

Supplementary Table 2. Comparing the characteristics of reference models and CNN approximations. The adaptation properties that describe each analytical model were estimated by computing the time needed for each respective response to fully decay from its maximum value (peak) after the stimulus onset to its steady state. A 1-kHz pure tone of 70 dB SPL was used for all auditory models and the responses at the corresponding CF were considered. Three durations are reported for the AN models, corresponding to the adaptation time courses of the HSR, MSR and LSR fibers respectively. The Hodgkin-Huxley neuron model can generate an infinite train of spikes for high stimulus levels (see Methods), hence the reported time corresponds to the length of the selected input window. For each CNN architecture, the RF lengths were computed from the selected N_{enc} and filter length parameters using Eq. 4 and were converted to ms durations (f_s = 20 kHz for all CNN models). Choosing architectures with longer RFs than the computed peak-to-steady-state durations resulted in models that accurately approximated the respective analytical descriptions. The filters per layer and total number of trainable parameters of each CNN model are also shown for comparison purposes.

	**Analytical model**	**CNN model**				
	peak-to-steady-state duration (ms)	Encoder layers (N_{enc})	Filter length	RF (ms)	Filters /Layer	Parameters
Verhulst et al. IHC[44]	25.45	3	16	5.3	128	1,317,505
Dierich et al. IHC[10]	63.85	3	16	5.3	128	1,317,505
Verhulst et al. AN[9]	349.95/716.25/846.45	14	8	5,734.1	64	1,250,177
Zilany et al. AN[58]	186.7/272.75/322.8	14	8	5,734.1	64	1,250,177
Hodgkin-Huxley[1]	102.4	9	16	383.3	64	1,511,489

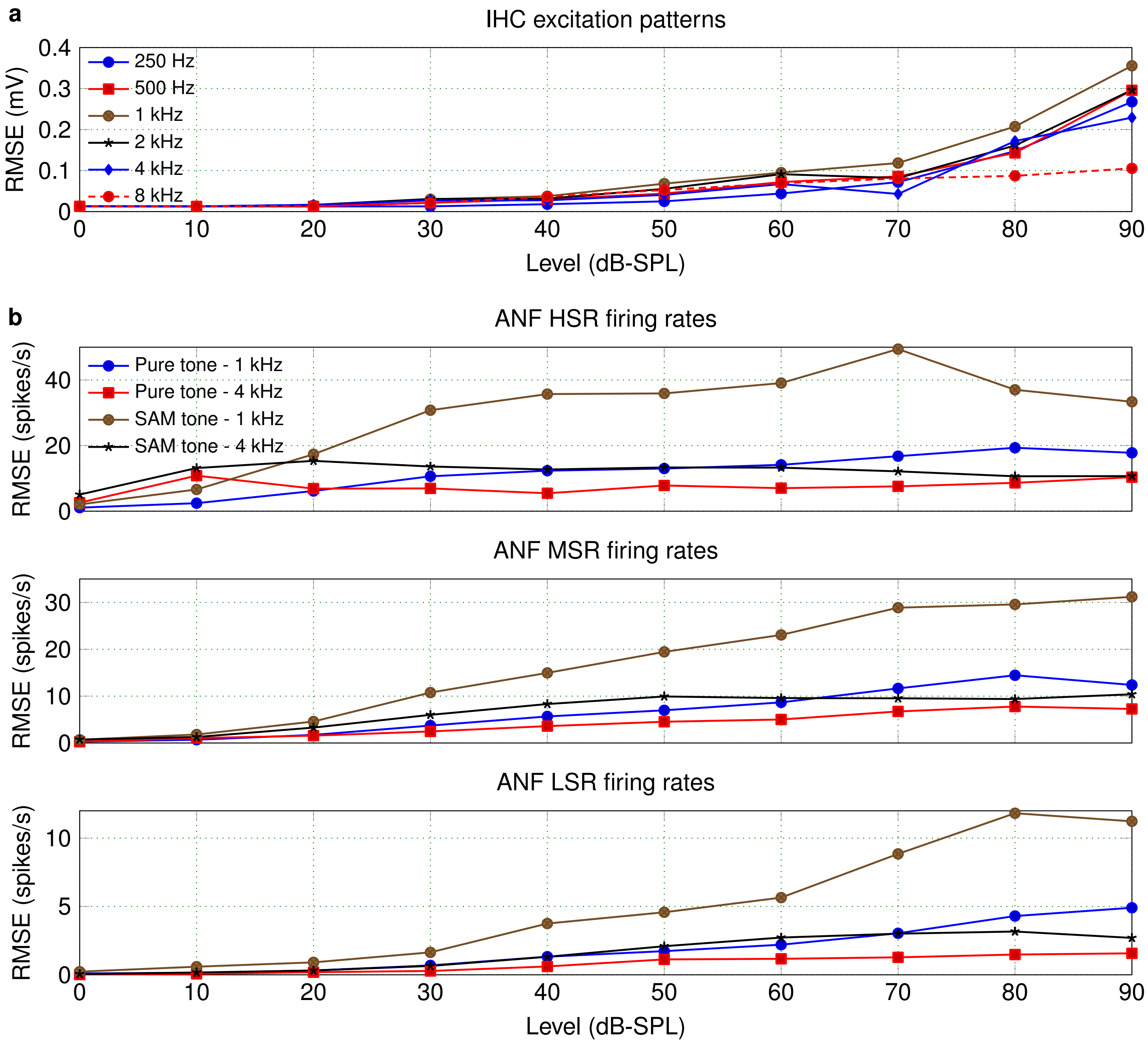

Supplementary Figure 5. RMSEs computed for the trained CNN approximations of the Dierich IHC and Zilany ANF models. a RMSE between simulated average IHC receptor potentials $\overline{V}_{\mathrm{m}}$ of the Dierich et al. IHC model[10] and the respective CNN approximation, computed across level for tonal stimuli of different frequencies. **b** RMSE between simulated ANF firing rates of the Zilany et al. ANF model[58] and the respective CNN approximation, computed for each of the tonal stimuli used for the ANF evaluation.

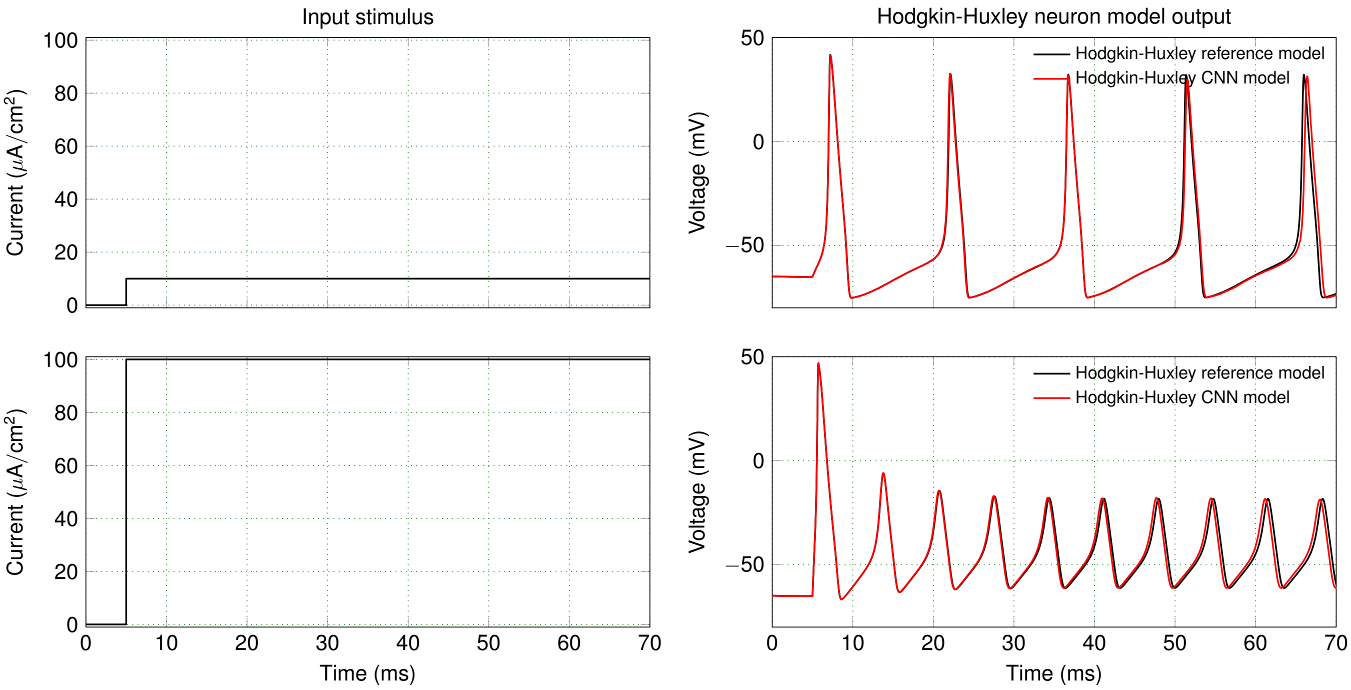

Supplementary Figure 6. Approximation of the standard Hodgkin-Huxley (HH) model. To demonstrate the extension of our framework to non-auditory models, we applied our methodology to the HH model[1] (see Methods for more details). The outputs of the trained CNN architecture and the original HH model are compared here for two different stimuli.
