# Supplementary material for: A convolutional neural-network framework for modelling auditory sensory cells and synapses
Source: Commun Biol. 2021 Jul 1;4:827. doi: 10.1038/s42003-021-02341-5 (PMC8249591; doi:10.1038/s42003-021-02341-5)
Supplement: Supplementary file 2 — Reporting Summary [file 42003_2021_2341_MOESM2_ESM.pdf]

nature research

Corresponding author(s): Fotios Drakopoulos

Last updated by author(s): Jun 1, 2021

Reporting Summary

Nature Research wishes to improve the reproducibility of the work that we publish. This form provides structure for consistency and transparency in reporting. For further information on Nature Research policies, see our Editorial Policies and the Editorial Policy Checklist.

Statistics

For all statistical analyses, confirm that the following items are present in the figure legend, table legend, main text, or Methods section.

n/a	Confirmed	
x	☐	The exact sample size (n) for each experimental group/condition, given as a discrete number and unit of measurement
x	☐	A statement on whether measurements were taken from distinct samples or whether the same sample was measured repeatedly
x	☐	The statistical test(s) used AND whether they are one- or two-sided<br>*Only common tests should be described solely by name; describe more complex techniques in the Methods section.*
x	☐	A description of all covariates tested
x	☐	A description of any assumptions or corrections, such as tests of normality and adjustment for multiple comparisons
x	☐	A full description of the statistical parameters including central tendency (e.g. means) or other basic estimates (e.g. regression coefficient) AND variation (e.g. standard deviation) or associated estimates of uncertainty (e.g. confidence intervals)
x	☐	For null hypothesis testing, the test statistic (e.g. F, t, r) with confidence intervals, effect sizes, degrees of freedom and P value noted<br>*Give P values as exact values whenever suitable.*
x	☐	For Bayesian analysis, information on the choice of priors and Markov chain Monte Carlo settings
x	☐	For hierarchical and complex designs, identification of the appropriate level for tests and full reporting of outcomes
x	☐	Estimates of effect sizes (e.g. Cohen's d, Pearson's r), indicating how they were calculated

Our web collection on statistics for biologists contains articles on many of the points above.

Software and code

Policy information about availability of computer code

Data collection	The code for running and evaluating the trained CNN models, including instructions of how to execute it, is available via https://doi.org/10.5281/zenodo.4889696 or https://github.com/HearingTechnology/CoNNear_IHC-ANF.
Data analysis	No data analysis software was used

For manuscripts utilizing custom algorithms or software that are central to the research but not yet described in published literature, software must be made available to editors and reviewers. We strongly encourage code deposition in a community repository (e.g. GitHub). See the Nature Research guidelines for submitting code & software for further information.

Data

Policy information about availability of data

All manuscripts must include a data availability statement. This statement should provide the following information, where applicable:

- Accession codes, unique identifiers, or web links for publicly available datasets
- A list of figures that have associated raw data
- A description of any restrictions on data availability

The source code of the auditory periphery model v1.1 used for training is available via https://doi.org/10.5281/zenodo.3717431 or https://github.com/HearingTechnology/Verhulstetal2018Model, the TIMIT speech corpus used for training can be found online via https://catalog.ldc.upenn.edu/LDC93S1. The source data underlying all graphs presented in the main and supplementary figures are available from the CoNNear IHC-ANF model repository https://github.com/HearingTechnology/CoNNear_IHC-ANF. Figures 2, 3, 5, 6 and 7 in this paper can be reproduced using this repository.
Figures 3 and 7 use data extracted from Figure 10 of "Palmer A, Russell I. Phase-locking in the cochlear nerve of the guinea-pig and its relation to the receptor potential of inner hair-cells. Hearing research. 1986;24(1):1–15"
Figures 6 and 7 use data extracted from Fig. 1 of "Winter IM, Palmer AR. Intensity coding in low-frequency auditory-nerve fibers of the guinea pig. The Journal of

the Acoustical Society of America. 1991;90(4):1958–1967", from Fig. 6 of "Taberner AM, Liberman MC. Response properties of single auditory nerve fibers in the mouse. Journal of neurophysiology. 2005;93(1):557–569" and from Figs. 5 and 8 of "Joris PX, Yin TC. Responses to amplitude-modulated tones in the auditory nerve of the cat. The Journal of the Acoustical Society of America. 1992;91(1):215–232"

Field-specific reporting

Please select the one below that is the best fit for your research. If you are not sure, read the appropriate sections before making your selection.

- [x] Life sciences
- [ ] Behavioural & social sciences
- [ ] Ecological, evolutionary & environmental sciences

For a reference copy of the document with all sections, see nature.com/documents/nr-reporting-summary-flat.pdf

Life sciences study design

All studies must disclose on these points even when the disclosure is negative.

Sample size	No experiments in study
Data exclusions	No experiments in study
Replication	No experiments in study
Randomization	No experiments in study
Blinding	No experiments in study

Reporting for specific materials, systems and methods

We require information from authors about some types of materials, experimental systems and methods used in many studies. Here, indicate whether each material, system or method listed is relevant to your study. If you are not sure if a list item applies to your research, read the appropriate section before selecting a response.

Materials & experimental systems

n/a	Involved in the study	
x		Antibodies
x		Eukaryotic cell lines
x		Palaeontology and archaeology
x		Animals and other organisms
x		Human research participants
x		Clinical data
x		Dual use research of concern

Methods

n/a	Involved in the study	
x		ChIP-seq
x		Flow cytometry
x		MRI-based neuroimaging
